# Effects of Diets with Different Energy Levels at Cold Temperatures on Gut Microbiota and Metabolic State in Growing–Finishing Pigs

**DOI:** 10.3390/microorganisms13092160

**Published:** 2025-09-16

**Authors:** Wei He, Guangdong Bai, Teng Teng, Baoming Shi, Li Wang

**Affiliations:** 1 State Key Laboratory of Swine and Poultry Breeding, Key Laboratory of Animal Nutrition and Feed Science in South China, Ministry of Agriculture and Rural Affairs, Institute of Animal Science, Guangdong Academy of Agricultural Sciences, Guangzhou 510640, China; hewei951011@163.com; 2 College of Animal Science and Technology, Southwest University, Chongqing 400715, China; baiguangdong@swu.edu.cn; 3 College of Animal Science and Technology, Northeast Agricultural University, Harbin 150030, China; tengteng@neau.edu.cn

**Keywords:** microbiota, metabolism, cold temperature, oil, high-energy, pigs

## Abstract

In cold-temperature regions, particularly on family farms, threats to livestock health constrain the potential of livestock husbandry. This study aimed to explore the effects of different dietary energy levels, adjusted by oil addition, on gut microbiota and metabolic homeostasis at cold temperatures. Twenty-four healthy pigs were randomly divided into two groups and fed a basal diet (BD) or a basal diet supplemented with oil (OD, with net energy increased by 100 kcal/kg) for 103 days. The cold temperature and humidity were maintained at 14 ± 2 °C and 65 ± 10%, respectively. On day 103 of the experiment, six pigs per group (three barrows and three females) were slaughtered after an overnight fast for sample collection: colon, colonic contents, plasma, and liver. The results showed that dietary oil addition remodeled the gut microbiota, forming a healthier microbial community characterized by a higher abundance of *Paludibacter*, *Parabacteroides*, *Peptococcaceae*, and *UCG-008* and a lower abundance of *Actinomyces*, *Turicibacter*, *Staphylococcus*, *Megamonas*, *Fusobacterium*, and *Achromobacter* (*p* < 0.05). Consistently, dietary oil addition resulted in higher levels of short-chain fatty acids (isobutyrate and isovalerate) and the Claudin-1 protein in the colon (*p* < 0.05). Plasma analysis showed that dietary oil addition increased energy metabolism and decreased inflammation. This change was characterized by higher levels of glucocorticoid, citrate, corticosterone, taurodeoxycholic acid, and ascorbic acid and lower levels of *IL-6* and hypoxanthine (*p* < 0.05). Transcriptomic and protein expression results in the liver further indicated that dietary oil addition alleviated energy stress and apoptosis by modulating metabolic states at cold temperatures. In conclusion, dietary oil addition improved gut health at cold temperatures in growing–finishing pigs, which was inextricably linked to the remodeling of the gut microbiota and metabolic states.

## 1. Introduction

Cold temperatures can lead to gut microbiota dysbiosis, tissue damage, inflammatory responses, and apoptosis in mammals, which further inhibit growth [1,2,3,4]. Generally, the increased basal metabolism and health risks under cold temperature conditions force the host to require more energy to maintain body temperature and normal biological processes [5,6,7]. Consequently, identifying appropriate nutritional strategies to improve host gut health and metabolic state at cold temperatures is essential.

Beyond adaptive thermogenesis, the gut microbiota plays a crucial role in maintaining host metabolic homeostasis in rodent models exposed to cold temperatures [8,9]. The gut microbiota produces large amounts of small-molecule metabolites, and its dysbiosis can disrupt barrier integrity, thereby inducing inflammation and metabolic dysfunction [4,10,11,12,13]. Hence, maintaining gut barrier integrity is critical for host metabolic homeostasis. Studies reveal that cold temperatures remodel the gut microbiota, leading to the formation of a “cold microbiota.” These changes further alter liver gene expression and metabolite profiles, thereby maintaining glucose homeostasis and promoting thermogenesis [2,8,9,13]. As the central hub of energy metabolism, the liver determines substrate fate in response to the external environment and nutrition, thereby maintaining metabolic homeostasis [14]. Collectively, the gut-liver axis plays a vital role in orchestrating metabolic disorders induced by cold temperatures and facilitating rapid adaptation to environmental changes [13,15].

As an essential nutrients and energy sources, fatty acids are indispensable in the mammalian diet and are typically categorized as saturated fatty acids, polyunsaturated fatty acids (PUFA), and monounsaturated fatty acids based on the number of double bonds [16,17,18]. Recent studies recommend increasing the proportion of unsaturated fatty acids in the diet, shifting from saturated fatty acids to unsaturated fatty acids, especially PUFA [18,19,20,21]. As a plant-derived lipid, soybean oil is the most commonly utilized in livestock and poultry diets. Meanwhile, the n-6 PUFA abundant in soybean oil must be obtained through the diet for livestock and poultry [18]. Notably, n-6 PUFA exert beneficial effects on cholesterol levels, insulin sensitivity, antioxidant capacity, and gut microbiota composition [18,22,23,24]. These studies suggest that dietary supplementation with soybean oil as an energy source for pigs may offer potential advantages under cold temperature conditions. Here, we hypothesized that dietary oil addition could improve gut health of pigs by remodeling the gut microbiota and metabolic state. In this study, we evaluated the effect of diets with different energy levels at cold temperatures on gut microbiota and metabolic state in growing–finishing pigs using multiomics analysis.

## 2. Materials and Methods

This study was conducted according to the guidelines for the care and use of laboratory animals of the Northeast Agricultural University and approved by the Northeast Agricultural University Ethics Committee of Animal Science and Technology College (NEAU-2013).

### 2.1. Animals and Experimental Design

This study was conducted at the Acheng Experimental Base of Northeast Agricultural University. After a 7-day adaptation period (basal diet), a total of 24 pigs (Duroc × Landrace × Yorkshire) were randomly divided into one of two experimental groups with 12 pigs per group (six barrows and six females) based on initial body weight (BW, 25.66 ± 0.33 kg) and gender. (1) BD: basal diet, (2) OD: basal diet supplemented with soybean oil, net energy increased by 100 kcal/kg. The experiment lasted for 103 days. All diets were formulated to meet or exceed the nutritional requirements of pigs, based on the Chinese Feeding National Feeding Standard for Swine [25] and National Research Council (NRC, 2012) recommendations (Table 1). The ratios of crude protein, amino acids, calcium, and available phosphorus to dietary energy levels were fixed across all groups.

The pig house was cleaned and disinfected (glutaraldehyde) before pigs entered the feeding room. During the experiment, pigs were housed in an environmentally controlled pens at an ambient temperature of 14 ± 2 °C and 65 ± 10% relative humidity. A boiler was installed in the piggery to maintain a constant temperature. The temperature of the feeding room was recorded by an electronic thermometer (Renke Measurement and Control Technology Co., Ltd., Jinan, China) with a probe (one measurement every 5 min). Moreover, pigs were allowed access to feed and water ad libitum throughout the study.

### 2.2. Sample Collection

On day 103 of the experiment, six pigs per group (three barrows and three females) with average final BW (130.83 ± 2.61 kg) were euthanized by electrical stunning after an overnight fast (12 h). Blood was collected rapidly from the jugular vein with heparin tubes, centrifuged at 4 °C for 10 min (3000 rpm), and the plasma was separated, collected, and frozen at −80 °C for further analysis. Liver index was calculated as the ratio of liver weight to BW. Liver and colon tissues were harvested, washed with saline (0.9% sodium chloride), and samples were fixed 4% paraformaldehyde and stored at 4 °C. Colonic contents and liver tissue were collected rapidly into sterile tubes and stored at −80 °C.

### 2.3. Analysis of 16S rRNA Gene Sequences

Total bacterial DNA in colonic contents was isolated using fecal genome DNAextraction kit (AU46111-96, BioTeke, Beijing, China). The primers used were 341F: 5′-CCTACGGGNGGCWGCAG-3′ and 805R: 5′-GACTACHVGGGTATCTAATCC-3′ (V3–V4 region). PCR reaction conditions were carried out as described previously [26]. After amplification, the integrity of the PCR products were assessed using agarose gel with a concentration of 2% [27]. Next, Illumina Novaseq 6000 system (Illumina, San Diego, CA, USA) was used to sequence samples meeting the sequencing conditions. The sequenced raw reads were filtered and processed with software (Trimmomatic v0.33) to obtain clean reads. The final valid data were obtained after denoising and splicing the denoised double-end sequences using QIIME2 [28,29,30]. After determining the community composition of each sample, a species abundance table was generated using QIIME at different taxonomic levels. The principal component analysis (PCA) plot (95% confidence interval) visualized β-diversity results based on Unweighted UniFrac distance, and adonis multivariate analysis of variance (Adonis) was used to assess differences in beta diversity between groups.

### 2.4. Quantitative Detection of Short-Chain Fatty Acids (SCFAs)

SCFAs in colonic contents were quantitatively analyzed by gas chromatography. In brief, samples were dissolved in ultrapure water, stored at 4 °C for 24 h, and centrifuged at 4 °C for 10  min (6000  rpm). The supernatant sample was filtered through a filter membrane (0.22 μm), and metaphosphoric acid (25%, *w*/*v*) was slowly added to the supernatant at a volume ratio of 1:5. The liquid was centrifuged at 4 °C for 10 min (10,000  rpm), filtered through a filter membrane (0.22 μm), and analyzed by gas chromatography system [31].

### 2.5. Analysis of Histopathology and Immunofluorescence (IF)

Colon and liver samples were washed, embedded in paraffin, deparaffinized, sectioned, and stained with hematoxylin-eosin (HE) [32]. Stained slides were scanned using a VS120 slide scanner (Olympus, Tokyo, Japan), and multiple random fields per section were photographed.

Remaining colon samples were used for Claudin-1 IF staining with primary antibodies as previously described [33]. Three random fields per slide were visualized and quantified using a fluorescent microscope (Zeiss Axioplan 2, Jena, Germany). The anti-Claudin-1 antibody (A11530) was purchased from ABclonal (Wuhan, China).

### 2.6. Inflammatory Factor

According to the manufacturer′s instructions, interleukin (IL)-6, IL-10, tumor necrosis factor-α (TNF-α), and endotoxin in plasma were quantified by ELISA kits (mlbio, Shanghai, China) using a microplate reader (Labsystems Multiskan MS, Vantaa, Finland).

### 2.7. Untargeted Metabolomics Analysis

Untargeted metabolomic analysis of plasma was conducted as described previously [34]. Briefly, a system composed of ultra-high performance liquid chromatography (Waters, Milford, MA, USA) with a high-resolution mass spectrometer (Waters) was used to analyze metabolites. The collected data were processed by software (Progenesis QI v2.4) for peak extraction and alignment, etc. Spearman correlation analysis and PCA (95% confidence interval) were used to assess the repeatability of the samples within the group and quality control samples. Differential metabolites were screened using the criteria: fold change (FC) > 1.2 or FC < 1/1.2, *p* < 0.05, and variable importance in projection (VIP) > 1. A pathway was considered KEGG-enriched if x/n > y/N; if the *p*-value was further < 0.05, it was identified as significantly enriched. x: the number of differentially expressed metabolites associated with the pathway; y: the number of background (all) metabolites associated with the pathway; n: the number of differentially expressed metabolites annotated to the KEGG pathway; N: the number of background (all) metabolites annotated to the KEGG pathway.

### 2.8. Biochemical Analysis of Plasma

According to the manufacturer′s instructions, plasma concentrations of albumin (ALB), globulin (GLB), total protein (TP), glucose (GLU), total bile acid (TBA), urea, triglyceride (TG), total cholesterol (TC), high-density lipoprotein (HDL), and low-density lipoprotein (LDL) were analyzed by commercial kits (Sino-UK, Beijing, China) using an automated biochemical analyzer (Mindray BS-200, Shenzhen, China).

### 2.9. Analysis of Plasma Hormones

According to the manufacturer′s instructions, insulin (ML002341, sensitivity > 78.13 pg/mL), glucagon (ML404356, sensitivity > 0.157 ng/mL), glucocorticoid (GC, ML414168, sensitivity > 0.312 ng/mL), glucagon-like peptide-1 (GLP-1, ML600784, sensitivity > 1.56 pg/mL), leptin (ML002355, sensitivity > 15.6 pg/mL), and growth hormone (GH, ML002349, sensitivity > 78.13 pg/mL) were quantified in plasma by ELISA kits (mlbio, Shanghai, China) using a microplate reader (Labsystems Multiskan MS, Finland).

### 2.10. Transcriptomic Sequencing in the Liver

Transcriptomic analysis in the liver was performed as described previously [35]. Briefly, total RNA was extracted and purified using TRIzol reagent (ThermoFisher, Waltham, MA, USA) [36]. RNA integrity was assessed using an RNA NanoDrop ND-1000 (Wilmington, DE, USA) on the Bioanalyzer 2100 System (Agilent, Santa Clara, CA, USA). Next, cDNA was synthesized for the fragmented RNA, and RNA sequencing libraries were generated using NEBNext Ultra RNA Library Prep Kit for Illumina (New England Biolabs, Ipswich, MA, USA) following the manufacturer’s instructions. Genes with FC > 1.5 and *p* < 0.05 were considered significant.

### 2.11. Analysis of Glucose-Metabolizing Enzymes Activity in the Liver

According to the manufacturer′s instructions, acetyl-coenzyme A (A-CoA), citrate synthase (CS), pyruvate kinase (PK), pyruvate dehydrogenase (PDH), and pyruvate carboxylase (PC) were quantified in the liver by ELISA kits (mlbio, Shanghai, China) using a microplate reader (Labsystems Multiskan MS, Finland).

### 2.12. Western Blot Analysis

Liver sample (0.1 g) was added to 1 mL RIPA lysis buffer (Beyotime, Shanghai, China, P0013B) and 10 μL PMSF (Beyotime, P1045), then centrifuged for 10 min (4 °C, 12,000 r/min) after low-temperature grinding. The supernatant was mixed to SDS loading buffer (Beyotime, P0015L) at a ratio of 4:1 and denatured at 95 °C for 10 min. Proteins were separated by SDS-PAGE and transferred to a polyvinylidene fluoride (PVDF, Merck Millipore, Burlington, MA, USA) membrane. Next, PVDF membranes were blocked with 5% non-fat dry milk in TBST (37 °C, 2 h), incubated with primary antibodies (4 °C, 12 h), and then incubated in secondary antibody (37 °C, 1 h) after washing with TBST. The primary antibodies β-actin (ABclonal, AC026, 1:50,000), carnitine palmitoyltransferase 1 (CPT1A, ABclonal, A20746, 1:1000), adipose triglyceride lipase (ATGL, Wanleibio, WL05654, 1:500), fatty acid transport protein 1 (FATP1, ABclonal, A12847, 1:1000), Bcl2-associated X protein (Bax, ABclonal, A19684, 1:1000), B-cell lymphoma-2 (Bcl-2, Wanleibio, WL01556, 1:500), Caspase-3 (Wanleibio, WL02117, 1:1000) were used. Protein bands were detected using an Alpha Imager 2200 (Alpha Innotech Corporation, San Leandro, CA, USA) and an enhanced chemiluminescence assay kit (Beyotime Biotechnology, Shanghai, China).

### 2.13. Statistical Analysis

SPSS 27.0 (IBM-SPSS Inc., Chicago, IL, USA) and GraphPad Prism 8 (GraphPad Prism Inc., La Jolla, CA, USA) were utilized for statistical analyses and data visualization. Student’s T-test and the Mann–Whitney U-test were used for normally and non-normally distributed data, respectively. Values were presented as the means ± SEM, and *p* < 0.05 were considered statistically significant. Spearman’s correlation analysis was applied to determine correlation coefficient, and *p* < 0.05 was considered significant.

## 3. Results

### 3.1. Growth Performance

The growth performance results of pigs are shown in Table 2. The results indicated that there was no significant difference in the initial BW of the pigs (*p* > 0.05), while the final BW and average daily gain (ADG) in the OD group were significantly higher than those in the BD group (*p* < 0.05).

### 3.2. The Diversity and Composition of Colonic Microbiota

**Figure 1:**
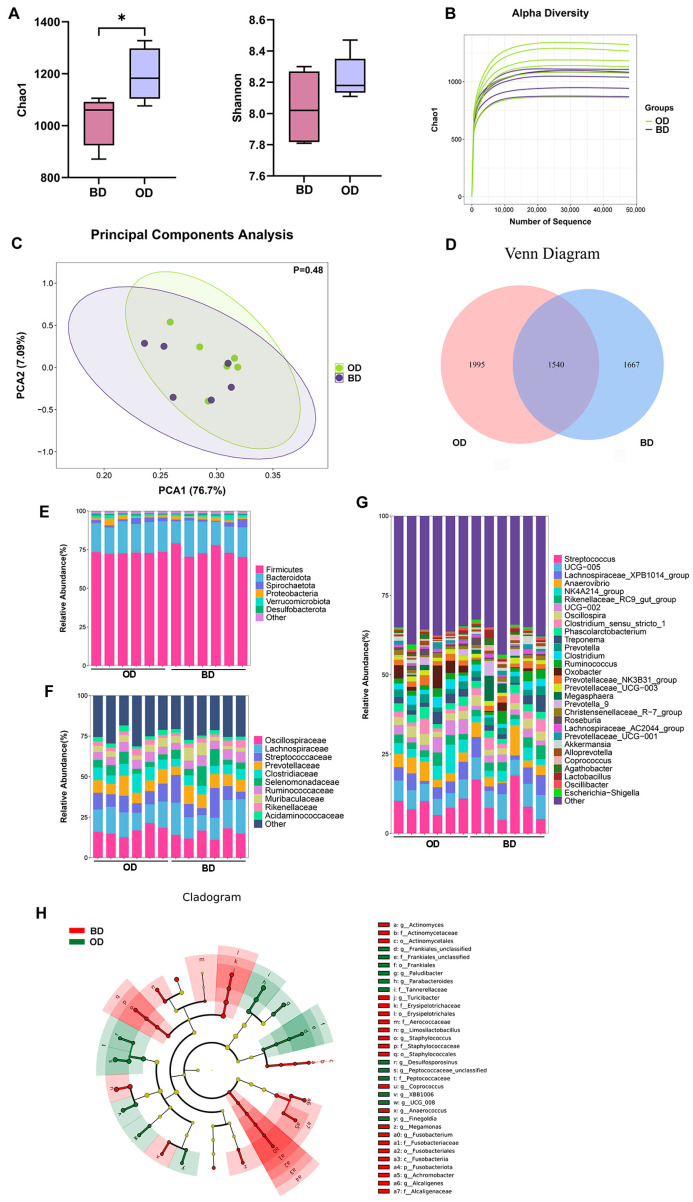
Colonic microbiota diversity and composition (*n* = 6). (**A**) The alpha diversity was measured using the Chao1 and Shannon indexes. (**B**) Rarefaction curves for the number of OTUs. (**C**) Principal component analysis (PCA) based on the Unweighted UniFrac distance. (**D**) Venn diagram for differential microbes in the comparisons BD vs. OD. (**E**) Relative abundance at the phylum levels (Top 6). (**F**) Relative abundance at the family levels (Top 10). (**G**) Relative abundance at the genus levels (Top 30). These figures were generated using R version 4.1.3. (**H**) Differentially abundant bacterial taxa were further identified by LEfSe (Linear discriminant analysis effect size) analysis. (*p* < 0.05, LDAscore > 2). The figure was generated using nsegata-lefse. BD: basal diet, OD: basal diet supplemented with soybean oil. Data are expressed as the mean ± SEM, * *p* < 0.05.

We assessed species alpha diversity using Chao1 and Shannon indexes across the samples. The Chao1 index was significantly higher in the OD group compared with the BD group (*p* < 0.05) (Figure 1A). Rarefaction curves for the number of Operational Taxonomic Units (OTUs) approached a plateau, indicating sufficient sequencing depth to cover OTUs present in colonic content samples (Figure 1B). PCA based on the Unweighted UniFrac distance showed that the microbiota composition was separated between the two groups (Figure 1C). Next, we further analyzed the differences in microbiota between the groups by VENN diagram (Figure 1D), and the BD and OD groups were assigned 1667 and 1995 OTUs in the colonic content samples, respectively.

Next, differences in colonic microbial composition were evaluated at various phylum, families, and genus levels (Figure 1E–H). *Firmicutes* and *Bacteroidota* were the two most abundant bacteria phyla, followed by *Spirochaetota* and *Proteobacteria* (Figure 1E). Compared with the BD group, the relative abundance of *Fusobacteriota* was lower in the OD group (Figure 1H). The most abundant families were *Oscillospiraceae*, *Lachnospiraceae*, *Streptococcaceae*, and *Prevotellaceae* (Figure 1F). The relative abundance of *Actinomycetaceae* (LDA 2.11), *Erysipelotrichaceae* (LDA 3.26), *Staphylococcaceae* (LDA 2.54), *Fusobacteriaceae* (LDA 2.12), and *Alcaligenaceae* (LDA 2.69) was lower in the OD group (Figure 1H). At the genus level, *Streptococcus* and *UCG-005* were the abundant bacterial genera in colonic content samples (Figure 1G). As shown in Figure 1H, the relative abundance of *Actinomyces* (LDA 2.11), *Turicibacter* (LDA 2.49), *Staphylococcus* (LDA 2.52), *Coprococcus* (LDA 3.21), *Anaerococcus* (LDA 2.04), *Megamonas* (LDA 2.22), *Fusobacterium* (LDA 2.12), *Achromobacter* (LDA 2.14), and *Alcaligenes* (LDA 2.58) was lower in the OD group compared with the BD group, while the relative abundance of *Paludibacter* (LDA 2.80), *Parabacteroides* (LDA 2.92), *Desulfosporosinus* (LDA 2.15), *Peptococcaceae* (LDA 3.38), and *UCG-008* (LDA 2.19) was higher in the OD group.

### 3.3. SCFAs Concentrations and Barrier Integrity in the Colon

**Figure 2:**
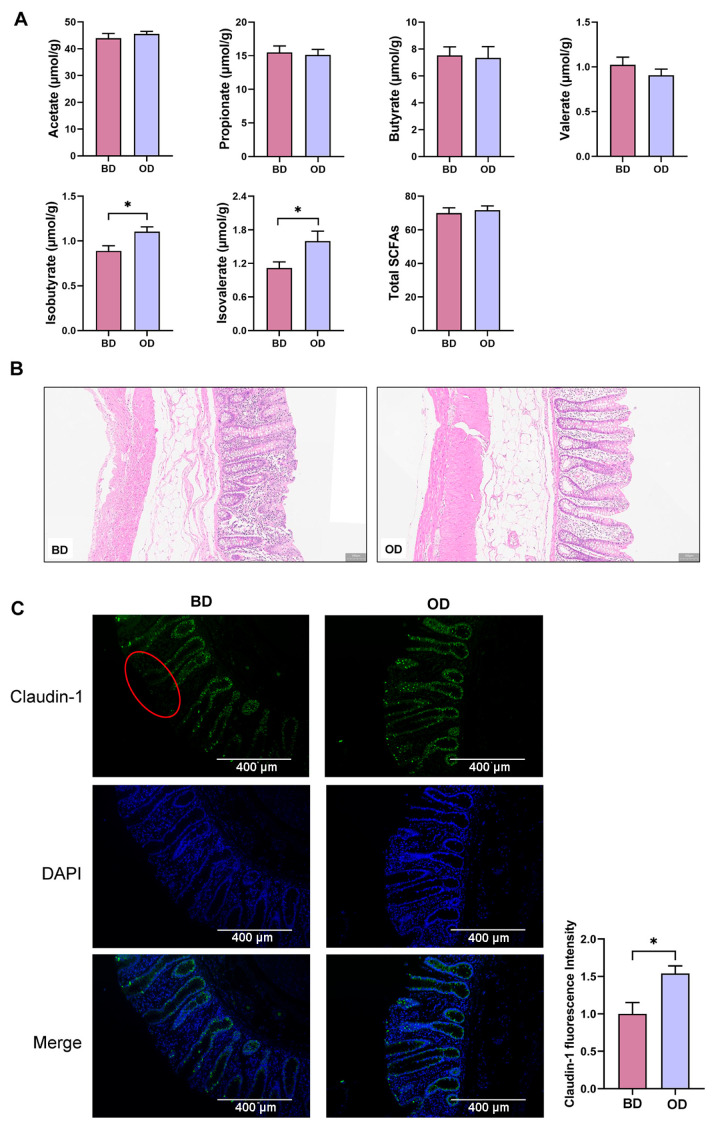
Short-chain fatty acid (SCFA) concentrations and barrier integrity of the colon (*n* = 6). (**A**) Concentrations of SCFAs in the colonic contents. (**B**) HE staining of the colon. (**C**) IF staining and analysis of Claudin-1 in the colon (*n* = 3). Red circles indicate barrier damage. IF, immunofluorescence. BD: basal diet, OD: basal diet supplemented with soybean oil. Data are expressed as the mean ± SEM, * *p* < 0.05.

For isobutyrate and isovalerate, higher levels in the colonic content was observed in the OD group compared with the BD group (*p* < 0.05). (Figure 2A). HE staining revealed mild damage and inflammatory infiltration in the mucosal layer of the colon in the BD group, which was not observed in the OD group (Figure 2B). Meanwhile, crypt atrophy of Lieberkuhn, a lower number of goblet cells and thinning of the muscle layer were observed in the BD group, whereas colonic integrity appeared better in the OD group. IF staining showed higher Claudin-1 fluorescence intensity in the OD group (*p* < 0.05) (Figure 2C).

### 3.4. Inflammatory Factors and Metabolites Composition in the Plasma

**Figure 3:**
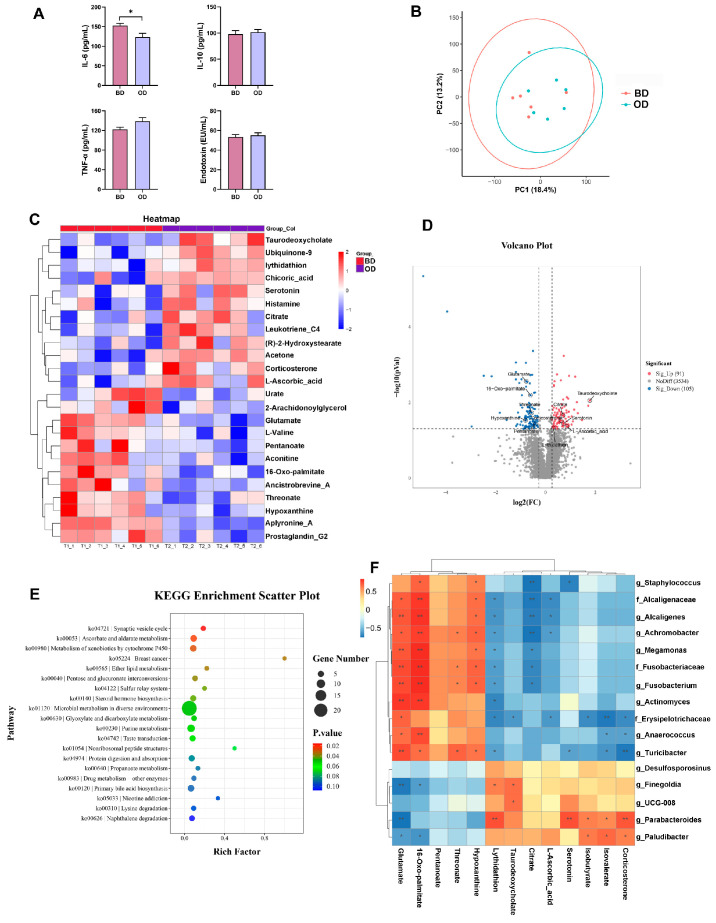
Analysis of hormones and metabolites of the plasma (*n* = 6). (**A**) Levels of inflammatory factors. (**B**) Principal component analysis (PCA) of samples. (**C**) Heatmap of the plasma metabolites. (**D**) Differential metabolites in the BD vs. OD comparisons. (**E**) KEGG pathway enrichment analysis of the differential metabolites. (**F**) Correlation analysis between the plasma metabolites and the colonic microbiota; red for positive correlations and blue for negative (* *p* < 0.05, ** *p* < 0.01). These figures were generated using R version 4.2.0. IL = Interleukin; TNF-α = tumor necrosis factor-α. BD: basal diet, OD: basal diet supplemented with soybean oil. Data are expressed as the mean ± SEM, * *p* < 0.05.

ELISA analysis showed that dietary oil addition decreased IL-6 levels (*p* < 0.05) (Figure 3A). To further understand the metabolic state between the two groups, an untargeted metabolomics analysis of plasma was performed. PCA indicating that dietary oil addition at cold temperatures altered the composition of plasma metabolites (Figure 3B). The relative abundance of some metabolites between the two groups is represented by a heatmap (Figure 3C). Analysis identified 3730 metabolites in the plasma, with 91 metabolites were upregulated and 105 metabolites were downregulated (Figure 3D). Compared with the BD group, the OD group exhibited higher levels of taurodeoxycholate (FC 3.43), citrate (FC 1.63), serotonin (FC 1.66), l-Ascorbic_acid (FC 1.82), lythidathion (FC 1.25), and corticosterone (FC 1.54), and lower levels of glutamate (FC 0.58), 16-oxo-palmitate (FC 0.66), threonate (FC 0.68), hypoxanthine (FC 0.57), and pentanoate (FC 0.75). To determine the functions of the changed metabolites, we performed Kyoto Encyclopedia of Genes and Genome (KEGG) pathway enrichment analysis. The enriched KEGG pathways (Top 20) were mainly associated with microbial metabolism in diverse environments, synaptic vesicle cycle, ascorbate and aldarate metabolism, ether lipid metabolism, pentose and glucuronate interconversions, steroid hormone biosynthesis, glyoxylate and dicarboxylate metabolism, purine metabolism, and primary bile acid biosynthesis (Figure 3E).

Next, we performed Spearman correlation analysis between colonic differential microbiota and plasma metabolites. As shown in Figure 3F, negative correlations were observed mainly between potentially harmful microbe *Erysipelotrichaceae*, *Anaerococcus*, and *Turicibacter* with isovalerate and isobutyrate, and positive correlations between *Parabacteroides* and *Paludibacter* with isovalerate and isobutyrate. In addition, negative correlations were observed mainly between lythidathion and citrate with a variety of potentially pathogenic bacteria. Notably, taurodeoxycholate was positively correlated with *Finegoldia* and *UCG-008*.

### 3.5. Biochemical Indicators and Hormones in the Plasma

As shown in Table 3, no significant differences were observed in biochemical indicators between the two groups (*p* > 0.05). For plasma hormones, higher levels of GC and GH were observed in the OD group compared to the BD group (*p* < 0.05).

### 3.6. Transcriptome and Correlation Analysis

**Figure 4:**
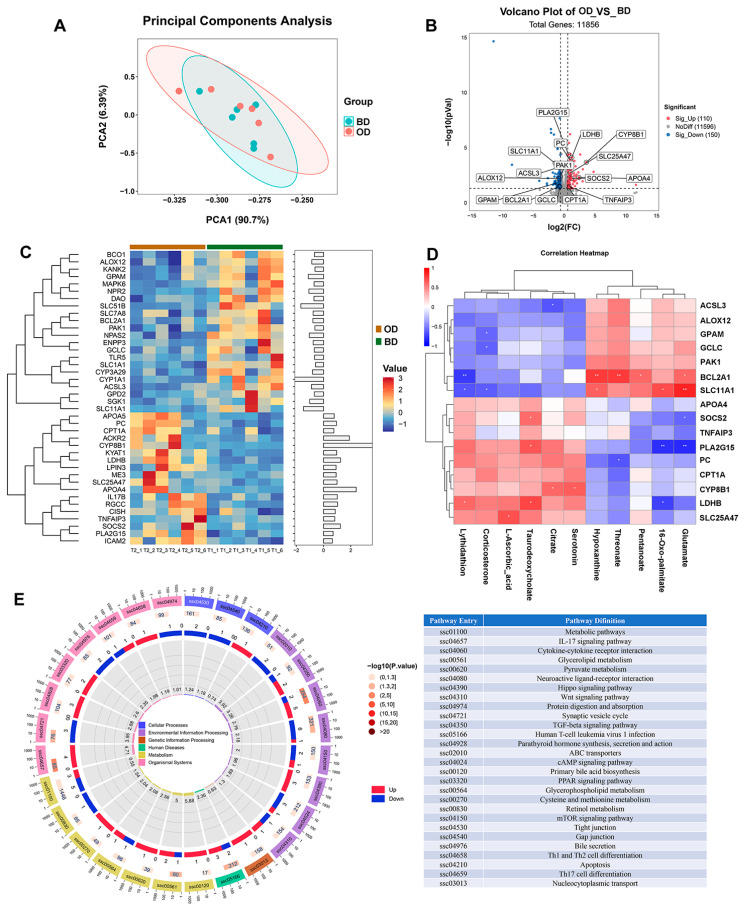
Transcriptomic analysis of the liver (*n* = 6). (**A**) Principal component analysis of samples. (**B**) Differentially expressed genes in the BD vs. OD comparisons. (**C**) Heatmap of the transcriptome analysis. (**D**) Correlation analysis between differentially expressed genes and plasma metabolites; red for positive correlations and blue for negative (* *p* < 0.05, ** *p* < 0.01). (**E**) KEGG pathway enrichment of differentially expressed genes. These figures were generated using R version 4.1.3. BD: basal diet, OD: basal diet supplemented with soybean oil.

We performed a transcriptome analysis in the liver, and PCA (95% confidence interval) showed distinct transcriptome profiles between different samples. The expression of some genes in each sample is presented by a heat map (Figure 4A). Analysis identified 1,1856 expressed genes, with 150 genes were downregulated and 110 genes were upregulated (Figure 4B,C). As shown in Figure 4B, volcano plot shows differentially expressed genes. Compared to the BD group, the OD group demonstrated higher expressions of lactate dehydrogenase B (LDHB, FC 2.37), sterol 12α-hydroxylase (CYP8B1, FC 12.15), pyruvate carboxylase (PC, FC 1.93), SLC25A47 (FC 1.99), CPT1A (FC 1.70), tumor necrosis factor-induced protein 3 (TNFAIP3, FC 1.78), while expressions of solute carrier family 11 member 1 (SLC11A1, FC 0.36), long chain acyl-CoA synthetase 3 (ACSL3, FC 0.54), glycerol-3-phosphate acyltransferase mitochondrial (GPAM, FC 0.46), BCL2-related protein A1 (BCL2A1, FC 0.58), and glutamate-cysteine ligase catalytic subunit (GCLC, FC 0.63) were lower. For KEGG pathway analysis, the major enrichment pathways for differential genes include metabolic pathways, cytokine-cytokine receptor interaction, IL-17 signaling pathway, protein digestion and absorption, PPAR signaling pathways, pyruvate metabolism, glycerophospholipid metabolism, and cysteine and methionine metabolism (Figure 4E).

Next, we performed a correlation analysis between differential genes and metabolites. As shown in Figure 4D, BCL2A1 and SLC11A1 were positively correlated with hypoxanthine (*p* = 0.005), threonate (*p* = 0.005), pentanoate (*p* = 0.037), and glutamate (*p* = 0.04), and negatively correlated with lythidathion (*p* = 0.008). Moreover, positive correlations were observed between taurodeoxycholate with LDHB (*p* = 0.010) and PLA2G15 (*p* = 0.020), and positive correlations between corticosterone with SLC11A1 (*p* = 0.032), GCLC (*p* = 0.037), and GPAM (*p* = 0.046). In addition, negative correlations were observed between PLA2G15 with 16-Oxo-palmitate (*p* = 0.003) and glutamate (*p* = 0.002), and positive correlations between CYP8B1 with citrate (*p* = 0.038) and serotonin (*p* = 0.025).

### 3.7. Glycolipid Metabolism and Apoptotic State of the Liver

**Figure 5:**
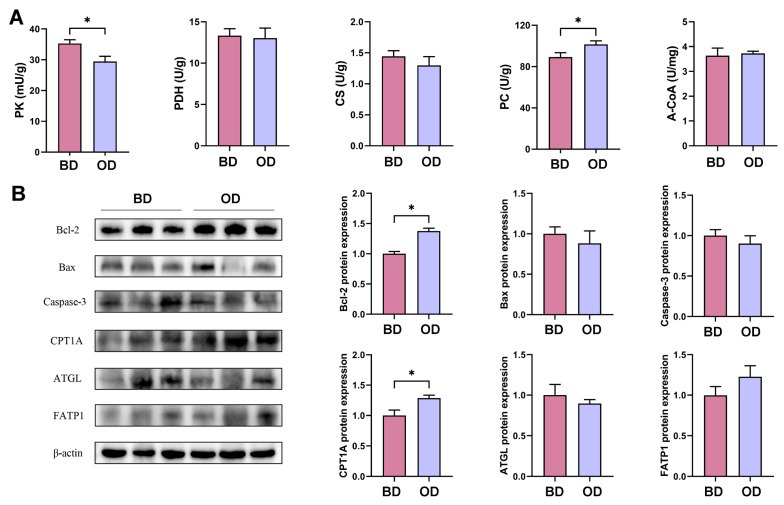
Analysis of glycolipid metabolism of the liver (*n* = 6). (**A**) The activity of enzymes related to glucose metabolism. (**B**) Apoptosis and lipid metabolism-related protein expression (*n* = 3). A-CoA = acetyl-coenzyme A; CS = citrate synthase; PC = pyruvate carboxylase; PDH = pyruvate dehydrogenase; PK = pyruvate kinase. BD: basal diet, OD: basal diet supplemented with soybean oil. Data are expressed as the mean ± SEM, * *p* < 0.05.

As shown in Figure 5A, ELISA analysis showed that dietary oil addition had lower PK activity at cold temperatures (*p* < 0.05), with no significant changes in PDH, CS, and A-COA between the two groups (*p* > 0.05). Surprisingly, dietary oil addition had higher PC activity, which was consistent with transcriptomic analysis (*p* < 0.05). As shown in Figure 5B, Western blot showed that the OD group had higher protein expression of CPT1A and BCL-2 in the liver, and lower protein expression of ATGL (*p* < 0.05).

### 3.8. Liver Index and Histopathology

**Figure 6:**
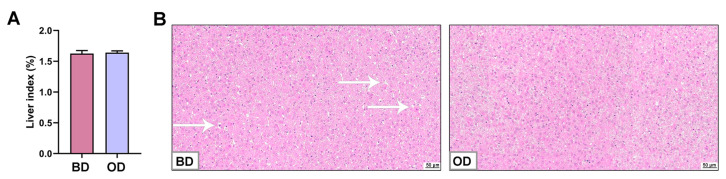
Analysis of histopathology of the liver (*n* = 6). (**A**) Liver index. (**B**) HE staining of the liver. White arrows indicate mild vacuolization. BD: basal diet, OD: basal diet supplemented with soybean oil.

No significant differences were observed in the liver index between the two groups (Figure 6A). Liver histopathology revealed no significant inflammation between the two groups (Figure 6B). Furthermore, mild vacuolization and damage occurred in the BD group, and a slight reduction in the number of nuclei in the BD group.

## 4. Discussion

Pigs are particularly sensitive to low temperatures due to a lack of brown fat, requiring more energy to maintain body temperature and normal biological processes [5,7]. Notably, the effects of differential dietary energy levels adjusted by oil addition on host gut microbiota and metabolites under prolonged cold temperatures are rarely illustrated. Based on the improved growth performance, we first assessed the alterations in colonic microbiota. The results showed that dietary oil addition alleviated the negative effects of cold temperature on the α-diversity in colonic microbiota. Meanwhile, we did not observe significant differences in the Shannon index, indicating that the dominant bacteria in the gut microbiota remained stable. Excitingly, analysis at the family level showed that dietary oil addition reduced the relative abundance of pathogenic bacteria associated with inflammation and intestinal damage [37,38,39]. Consistently, analysis at the genus level showed that dietary oil addition reduced the abundance of opportunistic pathogens. Specifically, the relative abundance of *Actinomyces*, *Turicibacter*, *Staphylococcus*, *Coprococcus*, *Anaerococcus*, *Megamonas*, *Fusobacterium*, *Achromobacter*, and *Alcaligenes* was significantly decreased in the OD group. An increase in the abundance of *Actinomyces*, *Turicibacter*, *Staphylococcus*, *Megamonas*, *Fusobacterium*, and *Achromobacter* is associated with gut microbiota dysbiosis and colitis [37,39,40,41]. In contrast, *Paludibacter* and *Parabacteroides* can produce various SCFAs, particularly acetate and propionate, which play a critical role in the regulation of host glucose homeostasis, gut integrity, and immune functions [42,43,44]. Currently, research on UCG-008 is limited, primarily due to the challenges associated with its in vitro cultivation. Consistently, *UCG-008* has been shown to decompose dietary fibers and plant polysaccharides to produce propionate [45]. In addition to generating butyrate and propionate, *Peptococcaceae* can metabolize aromatic amino acids from the diet, thereby influencing host health [46]. Studies have indicated a positive correlation between the abundance of *Peptococcaceae* and BW gain in hosts, which is consistent with our findings [47]. These results indicate that dietary oil addition at cold temperatures enhances the growth performance of pigs by improving gut microbiota structure and metabolic homeostasis.

As major metabolite of the microbiota, SCFAs are involved in gut barrier maintenance and anti-inflammatory effects, and the results of the colonic microbiota suggested that cold temperatures may induce intestinal barrier damage and inflammation. Therefore, we further determined SCFAs levels, barrier function, and colon morphology. Consistent with the results of the gut microbiota, dietary oil addition increased isobutyrate and isovalerate levels and improved colonic barrier function. Translocation of gut microbiota and reduced concentrations of isobutyrate and isovalerate may be key factors in the barrier damage caused by cold temperatures. Further examination of colonic histopathology showed slight inflammatory damage and reduced goblet cell number at cold temperatures. Previous study have shown that a decrease in goblet cell number disrupts integrity of the intestinal barrier by decreasing mucin secretion, further inducing inflammation [48]. Furthermore, microbial metabolites interact with gut cells and enter the bloodstream, affecting the metabolism and immunity of the host. Consistently, the results of plasma inflammatory factors showed lower *IL-6* levels with dietary oil addition at cold temperatures. Collectively, dietary oil addition at cold temperatures improves barrier function and inflammatory response.

Gut barrier damage and microbiota dysbiosis may induce host metabolic dysfunction, so we further evaluated the metabolic state of growing–finishing pigs [49,50]. Glucose is prioritized when the host faces cold stress, and hormones are mobilized to regulate energy metabolism as cold exposure continues [13]. GC and GH can promote lipolysis, releasing fatty acids to provide energy for the body [51]. Higher levels of GC and GH were observed in the OD group, which may be attributed to the adequate lipid supply alleviating the energy negative balance state induced by cold temperatures, thereby enhancing the secretion and metabolic efficiency of these hormones. Concurrently, the improved growth performance also support this observation. In addition, we observed no significant differences in plasma biochemistry, which may be due to we measured static plasma biochemical parameters and may not be able to capture subtle, dynamic metabolic changes. This study focused on long-term effects, and whether the short-term effects remain unchanged requires further investigation. Next, the analysis of plasma metabolites by untargeted metabolomics further validated the regulatory effect of dietary oil addition on host metabolic homeostasis. We observed altered metabolite composition, characterized by the upregulation and downregulation of multiple metabolites. Consistent with GC results, dietary oil addition had higher levels of citrate and corticosterone, which indicated an enhancement of glucose metabolism [52,53]. At the same time, lower hypoxanthine and higher ascorbic acid were observed, which may account for the alleviation of the cold temperatures-induced inflammatory response [54,55]. Overall, these differential metabolites were primarily enriched with ascorbate and aldarate metabolism, ether lipid metabolism, steroid hormone biosynthesis, pentose and glucuronate interconversions, glyoxylate and dicarboxylate metabolism, purine metabolism, and primary bile acid biosynthesis, which are closely related to energy metabolism. Furthermore, the microbial metabolism in diverse environments pathway was enriched for the most metabolites, which emphasizes the correlation between metabolites and gut microbiota. Specifically, a variety of bacteria (*Staphylococcus*, *Alcaligenes*, *Megamonas*, *Fusobacterium*, and *Achromobacter*) associated with dysbiosis and colitis were positively correlated with hypoxanthine and negatively correlated with citrate and ascorbic acid [39,40,41]. In addition, *Parabacteroides* and *Plaudibacter*, associated with immunomodulation and SCFAs production, positively correlated with corticosterone, isobutyrate and isovalerate [42,43]. The importance of gut microbiota in regulating plasma metabolism has been reported in previous studies, which is consistent with our finding that dietary oil addition can regulate metabolic homeostasis in growing–finishing pigs through colonic microbiota at cold temperatures [49,50].

**Figure 7:**
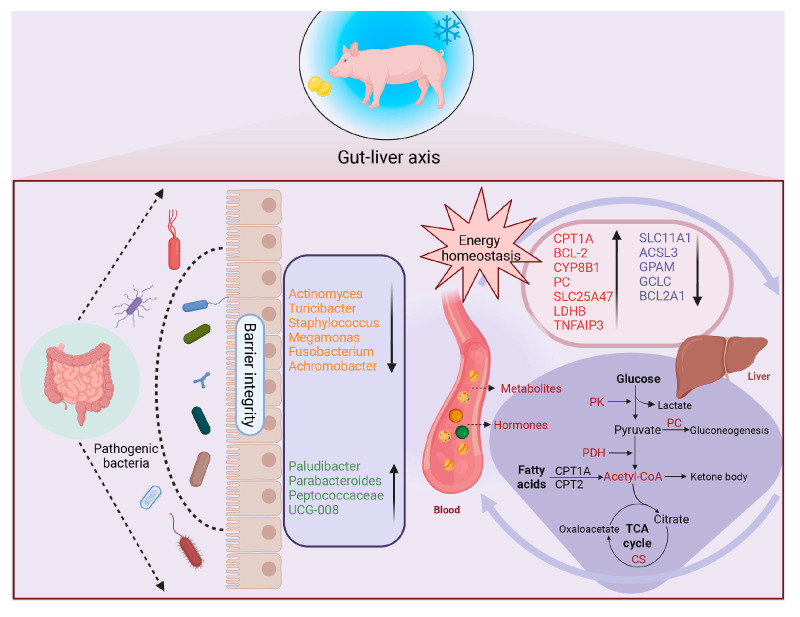
Multi-omics analysis reveals that dietary oil addition at cold temperature improves gut health and metabolic homeostasis by remodeling gut microbiota: A gut-liver axis study. The abundance of multiple potentially pathogenic bacteria was decreased, such as *Actinomyces*, *Turicibacter*, *Staphylococcus*, *Megamonas*, *Fusobacterium*, and *Achromobacter*. The abundance of multiple potentially beneficial microbes was increased, such as *Paludibacter*, *Parabacteroides*, *Peptococcaceae*, and *UCG-008*, which are mainly involved in immunomodulation and short-chain fatty acids (SCFAs) production. The remodeling of the gut microbiota structure further modulates host metabolic homeostasis via its metabolites, such as SCFAs. CPT1A, CYP8B1, PC, SLC25A47, LDHB, ACSL3, GPAM are related to energy metabolism. TNFAIP3 and SLC11A1 are related to inflammatory response. BCL-2, BCL2A1, GCLC are related to oxidative stress.

The liver, as the core organ regulating mammalian energy metabolism, determines substrate fate according to the external environment and nutrition [14]. Liver transcriptomic analysis revealed multiple differentially regulated genes after dietary oil addition at cold temperatures. CYP8B1 and CPT1A are involved in lipid absorption and transport, while LDHB, SLC25A47, and PC are closely related to glucose metabolism, consistent with enrichment pathway results [56,57,58]. Therefore, we further validated the glucose and lipid metabolism in the liver. Consistent with the transcriptomics results, Western Blot and ELISA assays indicated that dietary oil addition had higher protein expression of CPT1A and PC. The liver, receiving most blood supply from the portal vein, is continuously exposed to gut-derived microbial components and metabolites [59,60]. Consistently, LDHB, SLC25A47, and CYP8B1 were positively correlated with taurodeoxycholate, L-ascorbic acid, and citrate, respectively. Furthermore, high expression of GCLC and SLC11A1 indicated activation of Nrf2 antioxidant and inflammatory responses [61,62]. Likewise, as an anti-inflammatory factor, TNFAIP3 inhibits the activation of NF-κB and inflammasomes, helping maintain the integrity of epithelial cells [63,64,65]. These results suggest dietary oil addition can reverse inflammatory responses and energy stress induced by cold temperatures. As a member of the BCL-2 protein family, the mRNA expression of BCL2A1 was down-regulated in the OD group. However, further results for BCL-2 protein expression showed an opposite trend, which showed a positive effect on anti-apoptosis. This result may be due to post-transcriptional and post-translational regulation. Overall, there is a complex interaction of gut microbiota and metabolites with the liver, which is not limited to the metabolic regulation of nutrition. Increased plasma pro-inflammatory factors and colonic pathogens can induce liver injury, and dietary oil addition may alleviate the negative effects of cold temperature on the liver [66,67]. However, we observed an enrichment of multiple upregulated genes in the IL-17 signaling pathway, which may reveal that dietary oil addition may activate the IL-17 signaling pathway elicit inflammation in the liver. Conversely, liver index and histopathology analyses further indicated that dietary oil addition at cold temperatures had no effect on inflammatory damage, instead the restoration of mild vacuolization and the number of nuclei were observed. Therefore, the functional significance of IL-17 pathway upregulation requires further investigation. This study suggests that dietary oil addition mitigates the negative effects of cold temperatures on gut health by remodeling gut microbiota and metabolic state in growing–finishing pigs (Figure 7). In this study, the ADG increased by 0.10 kg/d, and the F/G decreased by 0.1. Although preliminary calculations indicate that feed costs are largely offset by the benefits of BW gain, this approach significantly reduces the time to market, thereby greatly enhancing pen turnover efficiency. Meanwhile, improvements in gut health are expected to reduce medical costs and mortality rates, thereby further increasing economic benefits. Therefore, we consider the diet evaluated in this study to be a viable and attractive nutritional strategy for growing–finishing pigs in cold climates. It should be noted that the major limitation of this study is the lack of a thermoneutral control group. Simultaneously, only three biological replicates were used in the protein analysis. Additionally, mucosal microbiota may be more closely associated with gut barrier integrity and metabolic functions, whereas the content sampling approach used in this study limited further exploration of the underlying mechanisms.

## 5. Conclusions

In summary, dietary oil addition enhanced the final BW and ADG of pigs. Furthermore, it improved the colonic microbiota composition and barrier function, accompanied by elevated levels of isobutyrate and isovalerate. Further results indicated that dietary oil addition maintained host metabolic homeostasis by regulating metabolite and hormone levels, which subsequently induced differential expression of liver genes related to energy metabolism and oxidative stress. This study provides new evidence for the interaction between oil addition diets and gut health at cold temperatures, offering new insights for dietary strategies in cold environments.

## Figures and Tables

**Table 1:** The compositions and ingredients of the diet.

Ingredients	Content (%)							
	d 1–29		d 29–52		d 52–76		d 76–103	
	BD	OD	BD	OD	BD	OD	BD	OD
Corn	69.15	64.25	73.40	68.60	79.35	74.85	81.95	84.25
Soybean meal	15.00	15.00	14.00	14.00	13.00	13.00	8.55	11.35
Full-fat soybean	10.45	13.32	8.49	11.25	4.20	6.75		
Wheat bran							6.00	
Soybean oil	1.28	3.23	0.50	2.47		1.90		0.91
Dicalcium phophate	1.29	1.36	0.98	1.03	0.82	0.86	0.62	0.75
Limestone	0.77	0.77	0.86	0.87	0.91	0.93	1.03	1.00
Salt	0.40	0.40	0.40	0.40	0.40	0.40	0.40	0.40
Lysine	0.42	0.42	0.28	0.27	0.25	0.24	0.34	0.28
Methionine	0.08	0.08	0.02	0.03	0.01	0.01	0.01	
Threonine	0.13	0.14	0.06	0.07	0.05	0.05	0.08	0.05
Choline chloride	0.03	0.03	0.01	0.01	0.01	0.01	0.02	0.01
Premix ^1^	1.00	1.00	1.00	1.00	1.00	1.00	1.00	1.00
Total	100.0	100.0	100.0	100.0	100.0	100.0	100.0	100.0
Nutrient levels ^2^								
Metabolizable energy, Mcal/kg	3.26	3.37	3.23	3.34	3.20	3.30	3.12	3.23
Ntet energy, Mcal/kg	2.53	2.63	2.51	2.61	2.49	2.59	2.45	2.54
Crude fat	4.97	6.38	4.05	4.80	3.70	4.38	2.47	3.10
Crude protein	17.58	18.41	16.37	17.41	15.27	15.73	13.44	13.75
Crude fiber	2.82	2.78	2.85	2.84	2.45	2.83	3.27	2.90
Lysine	0.99	1.03	0.83	0.87	0.72	0.75	0.61	0.64
Methionine	0.30	0.31	0.24	0.25	0.21	0.22	0.18	0.18
Threonine	0.61	0.64	0.52	0.55	0.46	0.48	0.39	0.41
Tryptophan	0.18	0.19	0.15	0.16	0.13	0.14	0.10	0.11
Calcium	0.65	0.67	0.60	0.63	0.57	0.60	0.55	0.57
Available phosphorus	0.28	0.29	0.22	0.23	0.20	0.20	0.17	0.18
Sodium	0.17	0.17	0.17	0.17	0.17	0.17	0.17	0.17
Chlorine	0.27	0.27	0.28	0.27	0.28	0.27	0.28	0.28

^1^ Provided the following per kilogram of diet: Fe, 140 mg; Cu, 25 mg; Mn, 35 mg; Zn, 80 mg; Se, 0.4 mg; I, 0.5 mg; vitamin A, 10, 000 IU; vitamin D, 3, 3000 IU; vitamin E, 63 mg; vitamin K3, 3 mg; vitamin B1, 3 mg; vitamin B2, 9.6 mg; vitamin B6, 4.5 mg; vitamin B12, 0.04 mg; niacin, 36 mg; d-pantothenic acid, 30 mg; d-biotin, 0.24 mg; and folate, 1.8 mg. ^2^ Crude fat, crude protein and crude fiber were analyzed values, and the rest were calculated values.

**Table 2:** Effect of dietary oil addition on growth performance of growing–finishing pigs exposed to cold temperatures (*n* = 12).

Items	Treatments		SEM	*p*-Value
	BD	OD		
Initial BW (kg)	25.57	25.74	0.327	0.799
Final BW (kg)	125.06	136.02	2.609	0.031
ADG (kg/d)	0.97	1.07	0.025	0.029
ADFI (kg/d)	2.48	2.64	0.059	0.180
F/G	2.57	2.47	0.028	0.061

ADG = average daily gain; ADFI = average daily feed intake; BW = body weight; F/G = ADFI/ADG. BD: basal diet, OD: basal diet supplemented with soybean oil. Data are expressed as the mean ± SEM. *p* < 0.05 was considered statistically significant.

**Table 3:** Effect of dietary oil addition on plasma biochemical parameters and hormones of growing–finishing pigs exposed to cold temperatures (*n* = 6).

Items	Treatments		SEM	*p*-Value
	BD	OD		
TP (g/L)	62.35	63.38	0.918	0.597
ALB (g/L)	27.96	27.20	0.755	0.637
GLB (g/L)	34.39	36.19	0.967	0.377
GLU (mmol/L)	10.76	10.59	0.876	0.927
TBA (µmol/L)	51.75	62.63	6.656	0.440
Urea (µmol/L)	6.44	6.75	0.338	0.673
TC (mmol/L)	2.18	2.04	0.057	0.219
TG (mmol/L)	0.63	0.54	0.038	0.254
HDL (mmol/L)	0.78	0.66	0.074	0.301
LDL (mmol/L)	1.22	1.24	0.052	0.822
Insulin (mIU/L)	29.92	28.72	0.735	0.442
Glucagon (pg/mL)	394.96	391.21	8.426	0.836
GC (ng/mL)	25.25	29.23	0.965	0.031
GLP-1 (pmol/L)	12.39	13.01	0.351	0.401
Leptin (ng/mL)	10.52	9.72	0.322	0.228
GH (ng/mL)	22.46	24.94	0.515	0.007

TBA = total bile acid; TP = total protein; ALB = albumin; GLB = globulin; GLU = glucose; TC = total cholesterol; TG = triglycerides; HDL = high-density lipoprotein; LDL = low-density lipoprotein; GC = glucocorticoid; GH = growth hormone; GLP-1 = glucagon-like peptide-1. BD: basal diet, OD: basal diet supplemented with soybean oil. Data are expressed as the mean ± SEM. *p* < 0.05 was considered statistically significant.

## Data Availability

The raw sequence data reported in this paper have been deposited in the Genome Sequence Archive in National Genomics Data Center, Chinese Academy of Sciences (GSA: CRA028965) that are publicly accessible at https://ngdc.cncb.ac.cn/gsa (accessed on 14 August 2025).

## Abbreviations

A-CoA	acetyl-coenzyme A
ALB	albumin
BD	basal diet
BW	body weight
CS	citrate synthase
GC	glucocorticoid
GH	growth hormone
GLP-1	glucagon-like peptide-1
GLB	globulin
GLU	glucose
HDL	high-density lipoprotein
HE	hematoxylin-eosin
IF	immunofluorescence
IL	interleukin
LDL	low-density lipoprotein
OD	basal diet supplemented with soybean oil
PCA	principal component analysis
PUFA	polyunsaturated fatty acid
PC	pyruvate carboxylase
PDH	pyruvate dehydrogenase
PK	pyruvate kinase
TBA	total bile acid
TP	total protein
TC	total cholesterol
TG	triglycerides
SCFA	short-chain fatty acid
